# More Than Just a Spearhead: Diverse Functions of PAAR for Assembly and Delivery of Toxins of the Contractile Injection Systems

**DOI:** 10.1128/msystems.01386-21

**Published:** 2021-12-07

**Authors:** Hao-Yu Zheng, Liang Yang, Tao Dong

**Affiliations:** a State Key Laboratory of Microbial Metabolism, Joint International Research Laboratory of Metabolic and Developmental Sciences, School of Life Sciences and Biotechnology, Shanghai Jiao Tong University, Shanghai, China; b School of Medicine, Southern University of Science and Technology, Shenzhen, China; c Department of Immunology and Microbiology, School of Life Sciences, Southern University of Science and Technology, Shenzhen, China

**Keywords:** protein secretion, interspecies interaction, effector, PAAR, T6SS, protein secretion

## Abstract

The type VI secretion system (T6SS) belongs to the evolutionarily related group of contractile injection systems that employ a contractile outer sheath to inject a rigid spear-like inner tube into target bacterial and eukaryotic cells. The tip of the rigid tube is often decorated by a PAAR-repeat protein as a key structural component. Many members of the PAAR protein family can also have additional and diverse functions by serving as toxins for those with extended domains or as carriers for interacting toxins. A plethora of toxin modules or modules of unknown functions have been bioinformatically predicted to be associated with PAAR either as a fused domain or as an interacting partner, and yet only a small number of PAAR proteins have been studied, highlighting the exciting and dire need for future research to better understand the diverse PAAR-mediated functions.

## COMMENTARY

Microbes have to overcome not only the fierce competition from neighboring microbes in polymicrobial communities but also the formidable host defenses during infection. To meet the head-on challenges of their adversaries, microbes have evolved various forms of weapon-like mechanisms ranging from diffusible antimicrobial molecules capable of long-range effects to contact-dependent killing of their specific neighbors (1–5). Of these weapons, the type VI secretion system (T6SS) is one of the most effective that can deliver a plethora of toxic effectors into both prokaryotic and eukaryotic species (1, 6–8).

**FIG 1:**
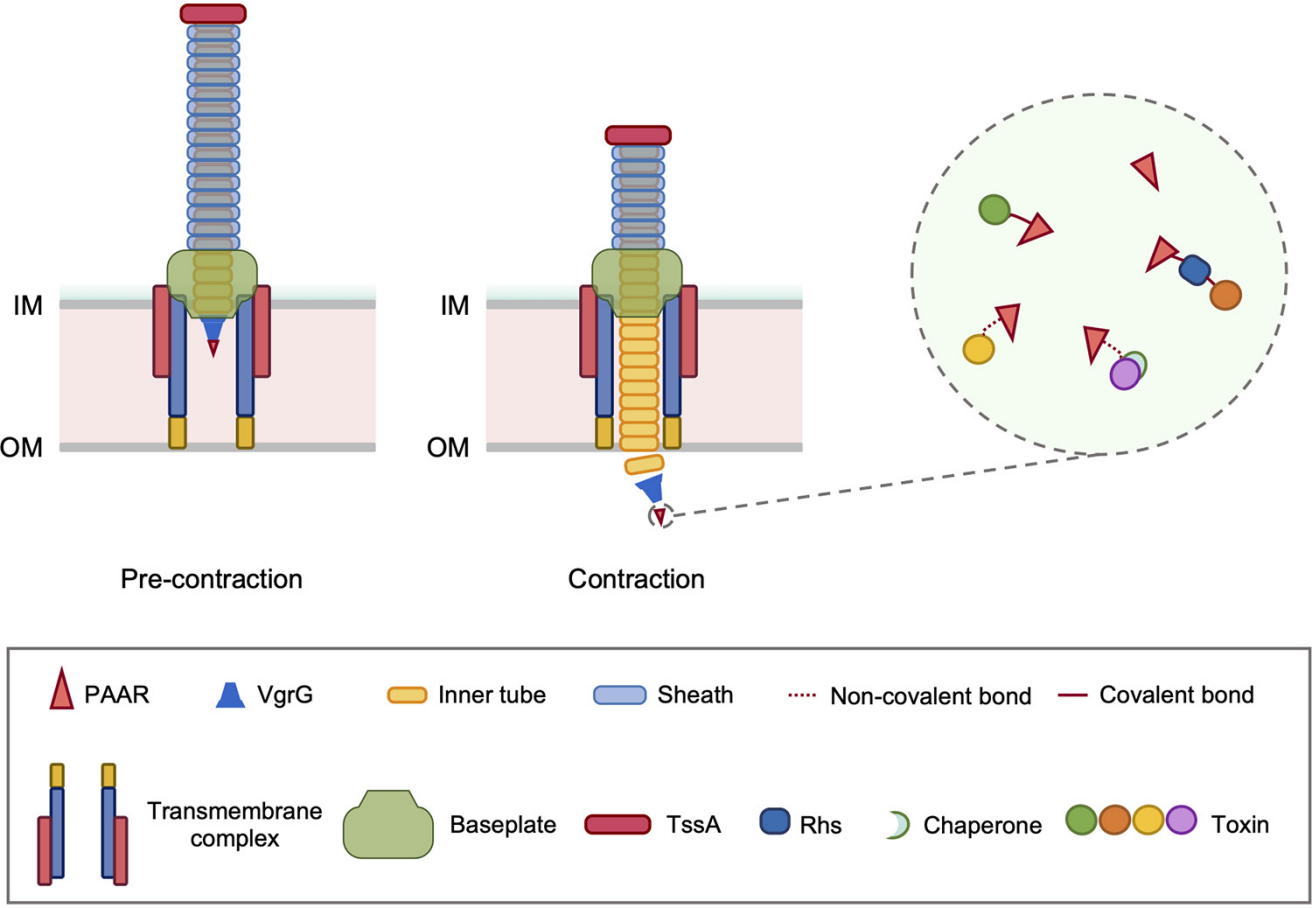
Schematic model depicting T6SS secretion. The rigid inner tube is wrapped around by the contractile outer sheath in a precontraction state. Upon sheath contraction, the inner tube and its tip spike complex consisting of VgrG-PAAR proteins are ejected out of the cell. The tip protein PAAR is highly diversified among T6SS species, whose homologs can be classified into several distinct classes, including PAAR-domain only, PAAR with a C-terminal tail that noncovalently binds to effectors and/or chaperones, extended PAAR with a C-terminal toxin domain, and extended PAAR-Rhs with or without a C-terminal toxin. IM, inner membrane; OM, outer membrane.

Based on structural similarity and protein conservation, the T6SS has been ascribed to a class of contractile injection systems (CIS) that also include the contractile phage tail, the R-type pyocin, the *Photorhabdus* virulence cassette, and the *Serratia* antifeeding prophage (Afp) (9, 10). They share a characteristic double-tubular structure with a rigid inner tube enclosed by an outer contractile sheath, and both tubes are made of stacking hexamers of their respective subunits (9). For the T6SS, the tube structure can span across the width, or less frequently the length, of a bacterial cell, and is anchored by a multicomponent baseplate and a transmembrane complex (Fig. 1) (11, 12). On the tip of the T6SS tube sits a spike complex composed of a VgrG-trimer and a cone-shaped PAAR which, together with the inner tube, are thrust out of the cell upon sheath contraction like a molecular spear. The released physical force is strong enough to enable the spear to puncture through the outer and inner membranes and the cell wall of Gram-negative cells (11, 13, 14). Excitingly, recent studies show that the T6SS spear can even penetrate and kill the relatively “tough” Gram-positive bacteria with much thicker cell envelopes (15, 16).

Because it is the effectors but not the physical puncture that kill bacteria (17, 18), the T6SS is not merely a spear but one smeared with deadly toxins. At the tip of the spear, PAAR-repeat proteins have been recognized as an important carrier for toxic effectors (19) or directly as toxins for those PAARs with extended N- or C-terminal domains (20, 21). A recent study has done an extensive search for PAAR homologs in the NCBI RefSeq database (22), and the results are consistent with previous findings (20). A greater number of PAAR proteins (47,625 homologs) have been found, and it is estimated that 23% of bacterial genomes encode at least one *PAAR* gene, which is in general agreement with the distribution of T6SS in bacteria. Although it is known that genomes commonly contain multiple copies of *PAAR* genes, it is astounding that 35 copies are found in the strain Chondromyces apiculatus DSM 436 (22). There is also a strong positive correlation between *PAAR* and *vgrG* genes. Having learned the 35 copies of *PAAR*, one should be less surprised that *C. apiculatus* DSM 436 also carries 54 copies of *vgrG* (22). In addition, there are 1,300 PAAR-associated toxin-encoding genes, of which 40% are PAAR proteins with extended domains and the majority are predicted nucleases (22).

Among the identified PAAR-associated toxins, there is an important group possessing the Rhs domain (20–25). Compared with the PAAR proteins, the Rhs proteins are more broadly distributed with homologs including many toxins found in both Gram-positive and Gram-negative bacteria as well as teneurins of eukaryotic species (23, 25–27). The PAAR-Rhs toxins are featured with an N-terminal PAAR domain, a middle Rhs domain, and a variable C-terminal toxin domain (16, 24, 28–30). Therefore, it is a highly interesting question how PAAR and Rhs domains are combined through evolution, and yet multiple biochemical evidences suggest that some, if not all, PAAR-Rhs toxins are subject to autocleavage at both the N terminus and the C terminus in several species, resulting in separated PAAR, Rhs, and toxin domains (16, 28, 30). Both the mechanism of cleavage and its biological significance remain to be elucidated.

Lastly, why is a PAAR protein required for T6SS assembly in some but not all species? For example, deletion of all three *PAAR* genes abolishes T6SS secretion in Acinetobacter baylyi ADP1 but not in Vibrio cholerae V52 or Aeromonas dhakensis SSU (20, 31). Recent studies on the importance of effector proteins may provide some hints. T6SS secretion in V. cholerae, *A. dhakensis*, Agrobacterium tumefaciens, and Enterobacter cloacae requires the presence of multiple VgrG-dependent effectors (17, 28, 31, 32). The previously known T6SS requirement for a heterotrimer VgrG spike in V. cholerae and in *A. dhakensis* is tightly linked to the specific structural effects of effectors because a pseudohomotrimer VgrG spike made of VgrG chimeras, differing only by the C-terminal effector-loading tail, is functional for T6SS assembly (33). There is also direct interaction between effectors and the baseplate protein TssK and the assembly chaperone TssA (33). These findings not only suggest that the effector stuffing within the baseplate cavity is crucial for stabilizing T6SS assembly in these species but also lead to the hypothesis that the differential requirement for PAAR in different species may be dependent on PAAR-associated effectors. Supporting this hypothesis is a recent observation that, although the PAAR domain can stabilize the trimeric VgrG, only the full length of PAAR-containing effectors with the bulky Rhs domain can support efficient T6SS assembly in E. cloacae (28). Given the prevalence of PAAR proteins, it is foreseeable that more discoveries are to be made in understanding PAAR biochemical activities and ecological impacts in diverse T6SS species and complex interspecies interactions.
